# Heterogeneous biological graph convolutional network for drug-target interaction prediction

**DOI:** 10.1371/journal.pone.0348895

**Published:** 2026-05-19

**Authors:** Haoran Zhu, Jianjia Wang, Zhen Hua, Chaoqun Wang, Zimu Zhang, Tong Yu, Ling Ge

**Affiliations:** 1 School of AI and Advanced Computing, Xi’an Jiaotong-Liverpool University, Suzhou, Jiangsu, China; 2 School of Computer Science and Informatics, University of Liverpool, Liverpool, United Kingdom; 3 School of Computer Engineering and Science, Shanghai University, Shanghai, Shanghai, China; 4 Department of Clinical Laboratory, Huaibei People’s Hospital, Huaibei, Anhui, China; Soochow University, CHINA

## Abstract

Drug–target interaction prediction plays a critical role in drug discovery by identifying potential therapeutic targets and elucidating underlying molecular mechanisms. However, existing computational methods generally rely on limited biological modalities and inadequately capture heterogeneous associations. To overcome these limitations, we propose a Heterogeneous Biological Graph Convolutional Network (HBGCN) that employs a hierarchical graph propagation architecture to integrate multimodal biological information and learn homogeneous and heterogeneous representations for drug–target interaction prediction. By incorporating both direct and indirect meta-paths, HBGCN captures complex relational dependencies among diverse biological entities. Experimental results demonstrate that HBGCN achieves competitive performance on benchmark datasets. Case studies indicate that HBGCN effectively identifies therapeutic drug candidates and reveals proteins and gene expression patterns associated with drug regulation. The source code and dataset are available at https://github.com/Saxon0918/HBGCN.

## Introduction

The prediction of drug–target interactions (DTIs) supports drug repositioning, adverse drug reaction detection, and molecular mechanism elucidation through the systematic analysis of binding patterns between bioactive compounds and targets [1]. Traditional DTI identification primarily relies on in vivo and in vitro experiments, including high-throughput screening and pharmacokinetic evaluation [2,3]. Although these approaches yield reliable results, they are typically constrained by high costs, substantial labor requirements, and limited scalability, particularly in large-scale studies. Consequently, computational approaches that exploit underlying biological characteristics have emerged as effective strategies for identifying potential drug–target interactions and improving the efficiency of drug discovery.

**Fig 1:**
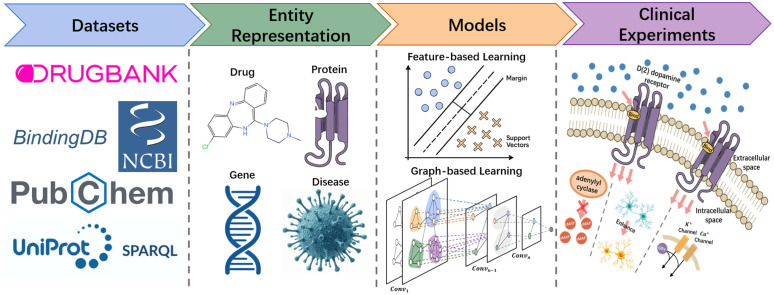
The workflow of DTI prediction, including four components: datasets, entity representation, models, and clinical experiments.

The general workflow of DTI prediction is illustrated in‌‌ Fig 1. Following the construction of a multimodal dataset, hierarchical molecular structures and known intra-entity interactions are extracted as initial features, which are subsequently refined through computational methods. Based on the strategies for information integration, existing DTI prediction studies are generally classified into feature-based and graph-based approaches.

Feature-based methods leverage machine learning and dimensionality reduction techniques to infer potential associations among biological entities. Probabilistic frameworks integrate heterogeneous similarity measures of drugs and targets within bipartite networks and employ probabilistic inference to improve prediction performance [4]. Low-rank matrix factorization techniques incorporate chemical structures, phenotypic profiles, and drug–drug interactions to uncover latent drug-disease associations [5]. Hierarchical clustering-based evaluation schemes mitigate biases in conventional data-splitting strategies and provide a biologically meaningful assessment of model generalizability [6]. In addition, some studies apply representation learning to derive high-dimensional feature vectors, which are subsequently used in random forest classifiers and Bayesian models for interaction prediction, while recent work further employs gradient boosting classifiers to improve representation discriminability [7–10]. Other studies employ random walk strategies, logistic regression, and ensemble learning to improve drug discovery [11–14].

Although machine learning approaches improve the efficiency of DTI prediction, their limited capacity to model subtle entity-specific features constrains predictive performance. In contrast, graph-based approaches integrate multimodal data by constructing heterogeneous networks, thereby revealing complex molecular interactions and functional associations. Some studies integrate attention mechanisms into graph neural networks (GNNs) to expand the receptive field, thereby capturing long-range dependencies among entities and uncovering potential DTI patterns [15–19]. Other studies leverage generative adversarial networks and contrastive learning strategies to improve embedding alignment across distinct entities, thereby enhancing prediction accuracy [20,21]. In addition, meta-learning frameworks that employ subgraph matching and weakly supervised information bottlenecks enhance predictive performance [22]. Recent work integrates higher- and lower-order biological information to characterize complex biological associations [23]. Furthermore, combining large language models for biological text representation with GNN-based structural encoding and knowledge graph reasoning further improves model robustness [24–26].

Despite recent advances in biological interaction prediction, existing studies exhibit limited performance and generalization capability when applied to sparse biological networks. Moreover, most approaches focus on modeling associations between specific pairs of entities and do not fully exploit the complex relationships inherent in biological systems. To address these limitations, we propose a graph convolutional network (GCN) framework that learns both homogeneous and heterogeneous representations of diverse biological entities, thereby improving the accuracy of DTI prediction. The main contributions are summarized as follows.

- We propose a Heterogeneous Biological Graph Convolutional Network (HBGCN) that hierarchically integrates multimodal information to enhance the representation learning of drugs and targets within a heterogeneous biological network.
- We construct a biological network dataset consisting of drugs, diseases, genes, and proteins to support DTI prediction.
- Comparative experiments with state-of-the-art algorithms demonstrate the effectiveness of HBGCN in drug repositioning, while case studies further validate its performance in identifying potential drug–protein pairings and gene regulatory mechanisms.

The remainder of this paper is organized as follows. Materials and methods provides a detailed description of the dataset construction, feature extraction, and the architecture of HBGCN. Results and discussion presents the implementation details and experimental results. Conclusion summarizes the key findings of this study and discusses potential directions for future research.

## Materials and methods

### Overview

**Fig 2:**
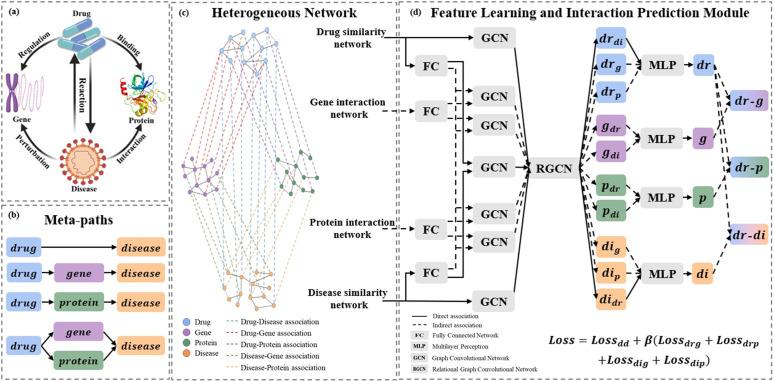
The workflow of the HBGCN. **(a)**: The associations between drugs and targets in the model. **(b)**: The four types of meta-paths between drugs and targets. **(c)** Construction of a heterogeneous drug-gene-protein-disease network by integrating multiple drug-related datasets. **(d)** The overall architecture of HBGCN, which consists of feature learning and interaction prediction modules.

In this section, we introduce the Heterogeneous Biological Graph Convolutional Network (HBGCN), which integrates multi-source biological data to predict drug-target interactions. As illustrated in Fig 2(a), the framework incorporates four fundamental types of biological entities, including drugs, genes, proteins, and diseases. To comprehensively capture complex associations, drug-target relationships are further classified into direct and indirect interactions. The HBGCN framework comprises three major components. The drug-target meta-path component leverages relational pathways to enhance biological interpretability. The similarity network integrates multiple similarity measures to refine interaction patterns among entities. The feature learning and interaction prediction module applies graph convolutional mechanisms to extract high-level representations and infer potential associations.

### Data acquisition and preprocessing

We construct a heterogeneous network consisting of four types of nodes and nine types of edges. Specifically, drug information is obtained from the DrugBank v3.0 database [27]. Motivated by existing drug network construction strategies [28,29], drug similarities are computed from structural fingerprints using the Jaccard similarity coefficient, which quantifies the proportion of maximal common substructures (*MCS*) shared between drug molecules [30]. Given two drug interaction graphs *dr*_1_ and *dr*_2_, the *JC*(*dr*_1_, *dr*_2_) is calculated as


$$\begin{array}{c} {JC(dr}_{1},{dr}_{2})=\frac{\left|MCS\left({dr}_{1},{dr}_{2}\right)\right|}{\left|{dr}_{1}+{dr}_{2}-MCS\left({dr}_{1},{dr}_{2}\right)\right|}. \end{array}$$ (1)

Disease entities are obtained from the Medical Subject Headings (MeSH) database, with similarities determined based on semantic relationships defined in the MeSH hierarchy, in which diseases are organized into coarse-grained parent and fine-grained child categories [31]. The semantic representation of each disease is derived from the cumulative semantic contributions of the disease itself and its related subcategories. Given two diseases *di*_1_ and *di*_2_, the similarity *S*(*di*_1_, *di*_2_) is calculated as


$$\begin{array}{c} {S(di}_{1},{di}_{2})=\frac{\left|CP\left({di}_{1},{di}_{2}\right)\right|}{\left|CP\left({di}_{1}\right)\right|+|CP\left({di}_{2}\right)|}, \end{array}$$ (2)

where CP(*) is the semantic contribution of diseases.

Gene and protein interaction networks are derived from HumanNet [32] and Human Protein Reference Database [33], respectively. Drug-disease and disease-protein associations are retrieved from the Comparative Toxicogenomics Database [34]. Drug-gene and drug-protein associations are collected from DGIdb v5.0 [35] and reference [1], respectively. Disease-gene associations are obtained from the DisGeNET v6.0 database [36].

Table 1 summarizes the number and density of nine types of relationships. The constructed dataset consists of 542 drugs, 394 diseases, 11,153 genes, and 1,512 proteins. Density is defined as the fraction of known associations among all possible pairs of the corresponding entity sets. Notably, disease-protein associations exhibit the highest density, indicating extensive connectivity, whereas drug-gene and drug-protein associations remain relatively sparse due to the limited number of known interactions. Although gene-gene interactions involve numerous connections, their density remains low due to the vast interaction space. These variations in density highlight the structural heterogeneity of the dataset and reflect the diverse nature of biological relationships.

**Table 1:** Data volume, comprising four entities, as well as nine homogeneous or heterogeneous connections.

Edge Type	Count	Density
Drug-drug similarity	12290	0.0418
Disease-disease similarity	29294	0.1887
Gene-gene interaction	1023439	0.0082
Protein-protein interaction	8284	0.0036
Drug-disease association	46669	0.2185
Drug-gene association	6716	0.0011
Drug-protein association	1516	0.0018
Disease-gene association	148903	0.0339
Disease-protein association	295070	0.4953

### Heterogeneous learning framework

This section introduces the proposed heterogeneous learning framework, which constructs a unified biological network over drugs, diseases, genes, and proteins by integrating meta-path semantics with entity similarity information. Building on this network, HBGCN is designed to capture relational dependencies among entities for identifying potential drug–target interactions.

Specifically, the meta-path is a predefined multi-hop schema among different entities, allowing models to capture higher-order interactions beyond direct associations. As illustrated in Fig 2(b), drug–disease associations are categorized into direct (*drdi*) and indirect associations. The indirect associations are further divided into three pathways, including drug-gene-disease (*drgdi*), drug-protein-disease (*drpdi*), and drug-gene-protein-disease (*drgpdi*). These meta-paths describe how drugs can be connected to diseases via intermediate entities, thereby encoding complex relational semantics.

The heterogeneous biological network constructed from entity similarities and meta-paths is shown in Fig 2(c). By extracting features from homogeneous and heterogeneous associations, we generate the initial vector representations of drugs (*V*_*dr*_), genes (*V*_*g*_), proteins (*V*_*p*_), and diseases (*V*_*di*_), which are subsequently input into the graph convolutional model.

The architecture of HBGCN is illustrated in Fig 2(d). The model achieves DTI prediction by progressively aggregating information from biological entities. To effectively extract features of homogeneous similarity networks of drugs and diseases, we individually propagate their initial representations through GCNs, which are calculated as


$$\begin{array}{c} x^{\left(l\right)}=\sigma \left({\overset{\sim }{D}}_{x}^{-\frac{1}{2}}{\overset{\sim }{A}}_{x}{\overset{\sim }{D}}_{x}^{-\frac{1}{2}}x^{\left(l-1\right)}W_{x}^{\left(l\right)}\right), \end{array}$$ (3)

where $x\in ({dr}_{1},{di}_{1})$, and $W_{x}^{\left(l\right)}$ is the weight matrix for the *l*^*th*^ graph convolutional layer. The normalized adjacency matrix is calculated by ${\overset{\sim }{A}}_{x}=A_{x}+I_{x}$, where *I*_*x*_ is the identity matrix. *A*_*x*_ is set to 1 if a known link exists between two entities; otherwise, it is set to 0. ${\overset{\sim }{D}}_{x}$ is the corresponding degree matrix, and σ(·) is the ReLU activation function. The inputs of the first layer ${dr}_{1}^{\left(0\right)}=V_{dr}$ and ${di}_{1}^{\left(0\right)}=V_{di}$. Incorporating neighboring information from homogeneous networks enables capturing local features of drugs and diseases, thereby improving the interpretation of relationships within a broader context.

In addition, fully connected (FC) layers are employed to refine the embedding representations of the four distinct entities within their respective homogeneous networks, which are calculated as


$$\begin{array}{c} x=\sigma \left(W_{x}V_{x}+b_{x}\right), \end{array}$$ (4)

where $x\in ({dr}_{2},g_{1},p_{1},{di}_{2})$; *W*_*x*_ and *b*_*x*_ represent the weight matrix and bias vector of each layer.

Different from homogeneous networks, where edges represent uniform relationships, heterogeneous networks involve various types of edges, each carrying distinct semantic information. To capture direct characteristics of drug-disease associations, we employ a GCN to learn the topological dependencies among nodes, calculated as


$$\begin{array}{c} \left({dr}_{3}^{\left(l\right)},{di}_{3}^{\left(l\right)}\right)=\sigma \left({\overset{\sim }{D}}_{drdi}^{-\frac{1}{2}}{\overset{\sim }{A}}_{drdi}{\overset{\sim }{D}}_{drdi}^{-\frac{1}{2}}\left({dr}_{3}^{\left(l-1\right)},{di}_{3}^{\left(l-1\right)}\right)W_{drdi}^{\left(l\right)}\right), \end{array}$$ (5)

where the inputs of the first layer ${dr}_{3}^{\left(0\right)}={dr}_{2}$ and ${di}_{3}^{\left(0\right)}={di}_{2}$; σ(·) is the ReLU activation function; $W_{drdi}^{\left(l\right)}$ is a layer-specific trainable weight matrix; ${\overset{\sim }{D}}_{drdi}=\left|\begin{array}{cc} {\overset{\sim }{D}}_{dr} & 0\\ 0 & {\overset{\sim }{D}}_{di} \end{array}\right|$ and ${\overset{\sim }{A}}_{dd}=\left|\begin{array}{cc} I_{dr} & A_{drdi}\\ A_{drdi}^{T} & I_{di} \end{array}\right|$.

In contrast, for indirect associations, we separately establish heterogeneous GCNs among various biological entities to explore potential latent relationships. Specifically, indirect GCNs are defined as


$$\begin{array}{c} \left(x^{\left(l\right)},y^{\left(l\right)}\right)=\sigma \left({\tilde{D}}_{xy}^{-\frac{1}{2}}{\tilde{A}}_{xy}{\tilde{D}}_{xy}^{-\frac{1}{2}}\left(x^{\left(l-1\right)},y^{\left(l-1\right)}\right)W_{xy}^{\left(l\right)}\right), \end{array}$$ (6)

where $\left(x,y)\in (\left(dr_{4},g_{2}),(dr_{5},p_{2}),(di_{4},g_{3}),(di_{5},p_{3}\right)\right)$; the inputs of the first layer ${dr}_{4}^{\left(0\right)}={dr}_{5}^{\left(0\right)}={dr}_{2}$, ${di}_{4}^{\left(0\right)}={di}_{5}^{\left(0\right)}={di}_{2}$, $g_{2}^{\left(0\right)}=g_{3}^{\left(0\right)}=g_{1}$, and $p_{2}^{\left(0\right)}=p_{3}^{\left(0\right)}=p_{1}$; σ(·) is the ReLU activation function; the definitions of ${\overset{\sim }{D}}_{xy}$, ${\overset{\sim }{A}}_{xy}$, and $W_{xy}^{\left(l\right)}$ are similar to the corresponding symbol definitions in Eq 5.

To capture global features of entities within the heterogeneous network, we employ a relational graph convolutional network (RGCN). Specifically, the representation of each node is updated by aggregating information from its neighbors, where corresponding weights are determined by the types of surrounding nodes and the relationships between them. As a result, RGCN can uncover valuable relational patterns, thereby enhancing the learning ability and predictive performance of the framework. The specific operation is


$$\begin{array}{c} h_{i}^{\left(l\right)}=\sigma \left(\sum_{r\in R}\sum_{j\in N_{i}^{r}}\frac{1}{\left|N_{i}^{r}\right|}W_{r}^{\left(l-1\right)}h_{j}^{\left(l-1\right)}+W_{s}^{\left(l-1\right)}h_{i}^{\left(l-1\right)}\right), \end{array}$$ (7)

where *R* is the number of edge types in the network; $N_{i}^{r}$ represents the set of neighbors of node *i* under edge type *r*; $W_{r}^{\left(l-1\right)}$ represents the weight parameter of edge type *r*; $W_{s}^{\left(l-1\right)}$ represents the weight parameter of node *i* itself; $h_{i}^{\left(l\right)}$ is the embedding of node *i* in the *l*^*th*^ RGCN layer, when *l* = 0, $h_{i}^{\left(0\right)}=\left({dr}_{1},{dr}_{2},{dr}_{3},{di}_{1},{di}_{2},{di}_{3},g_{2},g_{3},p_{2},p_{3}\right)$, and the output of the last layer of the RGCN model is $h_{i}=\left({dr}_{di},{dr}_{g},{dr}_{p},{di}_{dr},{di}_{g},{di}_{p},g_{dr},g_{di},p_{dr},p_{di}\right)$.

Finally, the updated representations of drugs, genes, proteins, and diseases are input into a multilayer perceptron (MLP) to transform the feature space and generate the final embeddings.


$$\begin{array}{c} x=W_{x}^{\left(l\right)}\left(\sum_{j\in 𝒩(x)}h_{j}\right)+b_{x}^{\left(l\right)}, \end{array}$$ (8)

where x∈(dr,g,p,di); 𝒩(x) denotes the set of entities associated with *x*; $W_{x}^{\left(l\right)}=\left\{W_{x}^{\left(1\right)},W_{x}^{\left(2\right)},W_{x}^{\left(3\right)}\right\}$ and $b_{x}^{\left(l\right)}=\left\{b_{x}^{\left(1\right)},b_{x}^{\left(2\right)},b_{x}^{\left(3\right)}\right\}$ are the weight and bias of the MLP. In addition, the activation function of each layer is ReLU.

### Interaction prediction

To quantify the interrelationships among biological entities, we define interaction scores based on the cosine similarity between their final embedding vectors. For example, the score between drugs and diseases is calculated as


$$\begin{array}{c} {\hat{S}}_{drdi}=dr\cdot {di}^{T}. \end{array}$$ (9)

Analogously, we derive the relational strengths for drug-gene and drug-protein associations. During training, to ensure that each type of entity effectively captures the structural characteristics of the heterogeneous network, we adopt Mean Squared Error (MSE) as the loss function, which is defined as


$$\begin{array}{c} Loss={Loss}_{drdi}+\beta \left({Loss}_{drg}+{Loss}_{drp}+{Loss}_{dig}+{Loss}_{dip}\right), \end{array}$$ (10)

where ${Loss}_{drdi}=MSE\left(A_{drdi},{\hat{S}}_{drdi}\right)=\frac{1}{2}{\left(A_{drdi}-{\hat{S}}_{drdi}\right)}^{2}$, and the definitions of the remaining four loss terms are analogous to *Loss*_*drdi*_; β is the weighting coefficient that balances the contributions of direct and indirect association losses.

Algorithm 1 illustrates the overall workflow of model training. During this phase, all trainable parameters are initialized with random values. The loss is calculated by propagating the input data through the network, followed by backpropagation to update model parameters and data representations.

**Algorithm 1 Training Procedure of HBGCN**

**Input:**

- Drug features *V*_*dr*_; disease features *V*_*di*_; gene features *V*_*g*_; protein features *V*_*p*_

- Drug-disease associations *A*_*drdi*_; drug-gene associations *A*_*drg*_; drug-protein associations *A*_*drp*_; disease-gene associations *A*_*dig*_; disease-protein associations *A*_*dip*_

- Training epochs *E*; pre-defined learning rate *lr*; weighting coefficient β

**Output:**

- Interaction scores between drugs and targets ${\hat{S}}_{drdi}$, ${\hat{S}}_{drg}$, and ${\hat{S}}_{drp}$

**Method:**

1: Randomly initialize the weight *W* and bias *b* of each layer.

2: **for** *epoch* = 1 to *E* **do**

3:  **Feature Learning module**

4:  Calculate homogeneous drug vector *dr*_1_ and disease *di*_1_ vector by Eq 3.

5:  Calculate *dr*_2_, *g*_1_, *p*_1_, and *di*_2_ by Eq 4.

6:  Calculate direct drug vector *dr*_3_ and disease vector *di*_3_ by Eq 5.

7:  Update *dr*_4_, *dr*_5_, *g*_2_, *g*_3_, *p*_2_, *p*_3_, *di*_4_, and *di*_5_ by Eq 6.

8:  Update entities vector by Eq 7

9:  Calculate final vector *dr*, *g*, *p*, and *di* by 8.

10.

11:  **Interaction prediction module**

12:  Calculate the interaction scores ${\hat{S}}_{drdi}$, ${\hat{S}}_{drg}$, and ${\hat{S}}_{drp}$ by Eq 9.

13: **end for**

## Results and discussion

In this section, we present the experimental settings and results. Extensive experiments are conducted to evaluate the performance of HBGCN, including comparisons of predictive accuracy with baseline methods, drug–target case studies, ablation studies, and hyperparameter analyses.

### Implementation details

In our implementation, we employ five-fold cross-validation, where each test set consists of a randomly selected subset of known positive drug-target interactions and an equal number of negative samples. The feature embedding dimensions for the FC, GCN, and RGCN modules are set to 256, while the output dimensions of the three-layer MLP are 256, 128, and 64, respectively. The model is trained for 2000 epochs using the Adam optimizer to minimize the loss function. The learning rate is 0.0001, and β in the loss function is 0.05. To evaluate predictive performance, we adopt standard metrics such as the area under the ROC curve (AUC), the area under the precision-recall curve (AUPR), F1-score, precision, and recall, which are commonly used in DTI prediction tasks.

### Comparison of predictive performance

To evaluate the predictive performance of HBGCN, we compare it with multiple state-of-the-art algorithms. Some of these methods incorporate graph-based learning with attention mechanisms and similarity-based matrix decomposition for DTI prediction, while others employ traditional methods such as collaborative filtering and low-dimensional vector projection. To ensure the rigor of comparative experiments, all models are trained and evaluated on the same benchmark dataset. Notably, for baselines that do not utilize all modalities, we provide only the required input data to avoid introducing unavailable information. The details of the baseline methods are summarized as follows.

- DMHGNN [37]: A double multi-view heterogeneous graph neural network that jointly learns from a heterogeneous network informed by meta-path semantics and drug–target pair graphs.
- GCNMM [38]: A graph convolutional network based on meta-paths and mutual information for DTI prediction.
- HMLKGAT [39]: A multi-layer graph model enhanced by adaptive attention mechanisms to capture associations among biological entities within a drug-protein-disease heterogeneous network.
- DRAGNN [16]: A weighted local information augmented graph neural network for drug repositioning.
- MGRMF [40]: A similarity-based approach utilizing low-rank matrix factorization with multi-graph regularization to enhance the accuracy of drug-disease association predictions.
- LBMFF [25]: A method that extracts the drug and disease fusion similarity matrix through BERT and refines feature embedding using a graph convolutional network.
- DRWBNCF [41]: A neural collaborative filtering framework designed to infer novel potential drugs for diseases.
- REDDA [42]: A heterogeneous graph neural network model that integrates multiple biological relationships.
- LAGCN [43]: A layer-wise attention graph convolutional network to effectively capture hierarchical drug-disease associations.
- MGRNNM [44]: A model that predicts the interactions between drugs and target proteins by integrating similarity measures and interaction patterns.
- DTINet [1]: A drug-target interaction prediction method based on the feature-based low-dimensional vector projection scheme.

The experimental results for drug–target interaction prediction are presented in Table 2, where diseases serve as targets. Except for the AUC, which is slightly lower than that of DRWBNCF, HBGCN achieves the highest AUPR (0.9611), F1-score (0.8912), precision (0.8761), and recall (0.9072), outperforming the best baseline by 5.43%, 7.40%, 5.97%, and 8.92%, respectively. Compared with other graph convolutional models, HBGCN leverages both direct and indirect molecular interactions to integrate multimodal biomedical information, thereby enhancing DTI prediction performance. The superior recall demonstrates that HBGCN effectively reduces false negatives and improves sensitivity in identifying true positive associations. Furthermore, some methods rely on traditional matrix decomposition techniques, which, despite demonstrating effectiveness in capturing global structural patterns, exhibit limitations in refining feature representations through hierarchical information propagation.

**Table 2:** Comparative experimental results.

Models	AUC	AUPR	F1-score	Precision	Recall
DMHGNN	0.9410	0.8975	0.8021	0.7748	0.7850
GCNMM	0.8938	0.8055	0.7472	0.7204	0.7385
HMLKGAT	0.9397	0.8598	0.7632	0.7687	0.7578
DRAGNN	0.8745	0.6743	0.6356	0.5966	0.6801
MGRMF	0.9408	0.8485	0.7464	0.7422	0.7506
LBMFF	0.9474	0.8706	0.8016	0.7899	0.8136
DRWBNCF	**0.9631**	0.9068	0.8172	0.8164	0.8180
REDDA	0.9550	0.8841	0.7901	0.7770	0.8035
LAGCN	0.9514	0.6991	0.6479	0.6810	0.6178
MGRNNM	0.9002	0.7076	0.6594	0.5669	0.7878
DTINet	0.9030	0.9023	0.6587	0.7041	0.6158
HBGCN	0.9584	**0.9611**	**0.8912**	**0.8761**	**0.9072**

Bold indicates the best, underlined indicates the second best.

### Case study

#### Drug repositioning.

To evaluate the effectiveness of HBGCN, we conducted two case studies on drug repositioning for depressive disorder and autistic disorder. To ensure an unbiased assessment, all known associations related to the target diseases were removed from the training set. HBGCN was subsequently employed to estimate interaction scores between drugs and diseases, and the top ten potential therapeutic candidates for each disorder were identified. The predicted results were validated by exploring the PubMed database for supporting evidence in publications and clinical studies.

**Fig 3:**
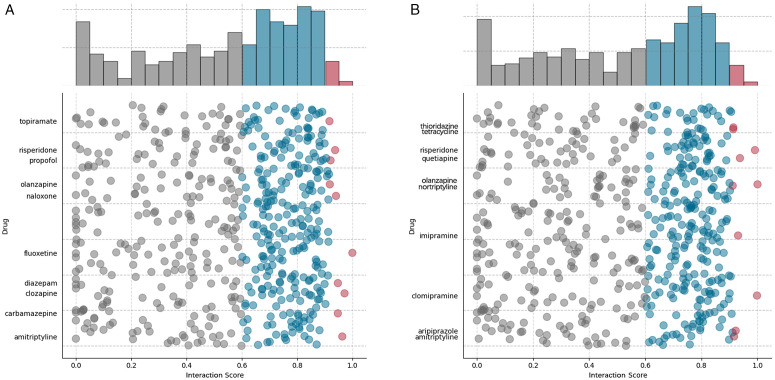
The distribution of interaction scores for drug repositioning. **(a)** The distribution of interaction scores for Depressive Disorder. **(b)**The distribution of interaction scores for Autistic Disorder. Red dots indicate the top ten most important drugs, grey dots represent drugs with a standardized score below 0.6, and blue dots denote drugs with moderate scores.

Depressive disorder is a mental health condition characterized by disturbances in mood, sleep, appetite, and cognitive function. The top ten candidate drugs predicted by HBGCN for the treatment of depressive disorder are shown in Fig 3(a) and Table 3. The results show that several predicted drugs have been reported in the literature as therapeutic agents for depressive disorder, primarily including selective serotonin reuptake inhibitors (SSRIs), antipsychotics, and mood stabilizers, such as fluoxetine, clozapine, amitriptyline, diazepam, carbamazepine, risperidone, and olanzapine. In contrast, other candidates, such as propofol and topiramate, have not yet been formally confirmed for the treatment of depressive disorder.

**Table 3:** Top ten predicted drugs for depressive disorder.

Rank	Candidate drugs	DrugBank ID	Evidences(PMID)
1	fluoxetine	DB00472	7623609, 7746839, 15650497, 2148340
2	clozapine	DB00363	28927482, 18203045, 34356654
3	amitriptyline	DB00321	12804503, 33438398, 7963067, 3897204
4	diazepam	DB00829	11684140, 3057815
5	carbamazepine	DB00564	3912763, 2187656, 7386656
6	naloxone	DB01183	36519188, 10708814
7	risperidone	DB00734	21189367, 12544386, 35993319
8	propofol	DB00818	uncomfirmed
9	olanzapine	DB00334	34986373, 37595997
10	topiramate	DB00273	uncomfirmed

To further assess the biological plausibility of the predicted candidates, we examined existing pharmacological evidence reported in the literature. Specifically, fluoxetine is widely prescribed for depressive disorder by inhibiting the serotonin transporter [45]. Furthermore, its neuroplasticity-enhancing properties promote synaptic remodeling, potentially contributing to sustained therapeutic efficacy. Clozapine modulates glutamatergic and γ-aminobutyric acid signaling, enhances neuroplasticity and neurotrophic factor expression, and reduces neuroinflammatory responses [46]. Amitriptyline modulates monoaminergic neurotransmission to produce antidepressant effects [47]. As an established mood stabilizer, carbamazepine has been shown to reduce manic and depressive episodes by modulating voltage-gated sodium channels and neurotransmitter activity [48]. In addition, although the efficacy of propofol and topiramate in treating depressive disorder remains unconfirmed, pharmacological studies indicate that both agents enhance inhibitory neurotransmission, which may contribute to anxiolytic and antidepressant-like effects [49,50].

Autistic disorder is a neurodevelopmental condition characterized by impairments in social interaction, communication deficits, and repetitive behaviors. The top ten candidate drugs predicted for the treatment of autistic disorder are shown in Fig 3(b) and Table 4. The predicted candidates mainly include neuropsychiatric drugs, such as olanzapine, clomipramine, risperidone, quetiapine, imipramine, aripiprazole, amitriptyline, thioridazine, and nortriptyline. In addition, tetracycline is identified as a potential candidate, although its therapeutic association with autistic disorder has not yet been reported in the literature.

**Table 4:** Top ten predicted drugs for autistic disorder.

Rank	Candidate drugs	DrugBank ID	Evidences(PMID)
1	olanzapine	DB00334	11501687, 9735606, 40132094, 30422498
2	clomipramine	DB01242	8498878, 1536276, 8919717, 1644740
3	risperidone	DB00734	17927305, 18278980, 26262903, 27409138
4	quetiapine	DB01224	21996644, 24434185, 25912535, 17389666
5	imipramine	DB00458	6355189, 7403355
6	aripiprazole	DB01238	21663425, 24600266, 21500873, 19948625
7	amitriptyline	DB00321	35780567
8	thioridazine	DB00679	15798789, 19393386
9	tetracycline	DB00759	uncomfirmed
10	nortriptyline	DB00540	5338335

As the candidate with the highest interaction score, olanzapine is an atypical antipsychotic that modulates neurotransmission through antagonism of dopamine and serotonin receptors, thereby alleviating behavioral symptoms such as irritability and aggression [51]. Clomipramine modulates serotonin systems to alleviate obsessive–compulsive–like symptoms [52]. Risperidone improves repetitive, aggressive, and self-injurious behaviors in individuals with autistic disorder, with some limitations related to tolerability [53]. Quetiapine and aripiprazole, both second-generation antipsychotics, modulate dopaminergic and serotonergic neurotransmission to alleviate irritability and aggressive behaviors associated with the disorder [54]. Although tetracycline has not been confirmed for the treatment of autistic disorder, its derivative minocycline has been shown to alleviate autism-like behaviors in mice by inhibiting microglial activation, reducing neuroinflammation, and improving hippocampal neurogenesis [55].

These findings demonstrate that HBGCN effectively identifies pharmacologically relevant compounds for diseases, highlighting its potential application in drug repositioning.

#### Drug-gene interaction prediction.

**Fig 4:**
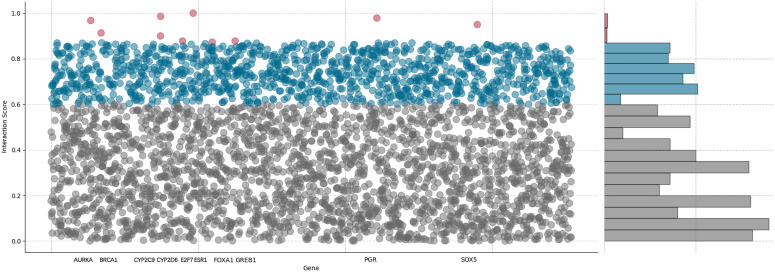
The distribution of drug-gene interaction prediction for Tamoxifen, with red dots representing the top ten important genes.

Tamoxifen is a selective estrogen receptor (ER) modulator that inhibits breast cancer progression by interfering with tumor growth signaling pathways. The top ten predicted genes associated with tamoxifen are shown ‌‌in Fig 4 and Table 5. The predicted genes mainly participate in estrogen receptor signaling, drug metabolism, cell cycle regulation, and tumor proliferation, such as *ESR1*, *PGR*, *GREB1*, *FOXA1*, *CYP2D6*, *CYP2C9*, *AURKA*, *BRCA1*, and *E2F7*. In contrast, the candidate gene *SOX5* has not yet been reported in the literature.

**Table 5:** Top ten predicted genes for Tamoxifen.

Rank	Candidate genes	NCBI ID	Evidences(PMID)
1	*ESR1*	2099	38868185, 26122181, 37697303, 33452979
2	*CYP2D6*	1565	29801584, 32270286, 22531359, 25618289
3	*PGR*	5241	10754487, 1634918, 19588487, 7427962
4	*AURKA*	6790	25362855, 29202611, 27461831, 37521867
5	*SOX5*	6660	uncomfirmed
6	*BRCA1*	672	37432545, 23918944, 11130383, 10696733
7	*CYP2C9*	1559	25940823, 27198207, 12207635
8	*GREB1*	9687	29212856, 33731348
9	*E2F7*	144455	30066905, 26397135
10	*FOXA1*	3169	36204307, 22476979, 27197147, 28270510

We further investigated existing functional and pharmacogenomic evidence to elucidate the regulatory mechanisms through which the predicted genes participate in tamoxifen response. As ER co-regulators, *ESR1*, *PGR*, *GREB1*, and *FOXA1* modulate the transcriptional response to tamoxifen and influence hormone receptor signaling pathways involved in breast cancer progression [56–58]. *CYP2D6* and *CYP2C9* are involved in the metabolic activation of tamoxifen [59], whereas *AURKA*, *BRCA1*, and *E2F7* play essential roles in regulating cancer cell proliferation, DNA damage response, and cell cycle progression [60,61]. Although the predicted association of *SOX5* remains unconfirmed, previous studies suggest that *SOX5* participates in apoptosis regulation, and its dysregulation may contribute to tumor development [62].

These findings indicate that the predicted genes are involved in multiple biological processes related to tamoxifen response, supporting the effectiveness of HBGCN in predicting drug-gene interactions.

#### Drug-protein interaction prediction.

**Fig 5:**
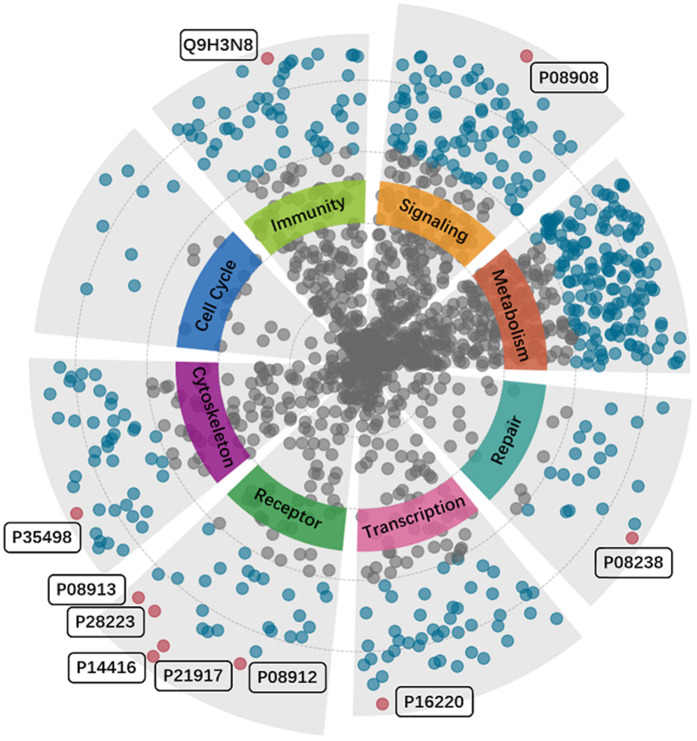
The distribution of drug-protein interaction predictions for Clozapine. The proteins are categorized into eight classes based on biological functions and involvement in physiological processes.

As shown in Fig 5, the predicted drug–protein interactions for clozapine are categorized into eight classes according to protein functions, biological pathways, and physiological processes. Notably, clozapine exhibits high binding affinity toward receptor proteins, which aligns with its pharmacological profile as an atypical antipsychotic that regulates synaptic transmission. Table 6 presents detailed information for the top ten protein targets. These targets primarily include neurotransmitter receptors and signaling-related proteins involved in dopaminergic, adrenergic, and cholinergic pathways.

**Table 6:** Top ten predicted proteins for Clozapine.

Rank	Candidate proteins	UniProtKB ID	Evidences(PMID)
1	D(2) dopamine receptor	P14416	11862380, 36980961, 2495973, 32976928
2	5-hydroxytryptamine receptor 1A	P08908	22012474, 12020849, 24423786, 29497362
3	D(4) dopamine receptor	P21917	28489950, 28347261, 23785734, 8064797
4	Cyclic AMP-responsive element-binding protein 1	P16220	uncomfirmed
5	Alpha-2A adrenergic receptor	P08913	28163420, 27956055, 25163438, 1971449
6	Histamine H4 receptor	Q9H3N8	27368152, 19413571
7	5-hydroxytryptamine receptor 2A	P28223	12020849, 30600145, 29497362, 11070178
8	Heat shock protein HSP 90-beta	P08238	uncomfirmed
9	Muscarinic acetylcholine receptor M5	P08912	17310388, 10328997, 10708730
10	Sodium channel protein type 1 subunit alpha	P35498	uncomfirmed

Specifically, dopamine receptors, including the D(2) and D(4) subtypes, are identified as major targets due to their involvement in modulating dopaminergic neurotransmission, which is central to alleviating the positive symptoms of schizophrenia [63]. Clozapine also interacts with 5-hydroxytryptamine receptors, contributing to the management of mood instability [64]. The Alpha-2A adrenergic receptor is critical for controlling norepinephrine release and mediating the sedative properties of the drug [65]. For unconfirmed targets, clozapine may promote neuroplasticity and cognitive enhancement by inhibiting sodium ion influx and molecular mechanisms [66,67].

These findings suggest that the predicted protein targets are associated with multiple neurotransmitter systems and signaling pathways related to clozapine pharmacology.

### Ablation study

To comprehensively evaluate the contribution of each component in HBGCN, we conduct an ablation study with three variants, each removing specific components.

- w/o protein: The first variant removes the protein component and relies solely on the drug-gene-disease heterogeneous network to learn embedding representations.
- w/o gene: The second variant relies solely on the drug-protein-disease heterogeneous network to learn embeddings.
- w/o indirect GCN: The third variant removes the indirect association GCN modules that encode multi-hop connectivity between drugs, diseases, and intermediate entities, while retaining the remaining components.

The results in Table 7 demonstrate that HBGCN achieves the best performance in drug candidate prediction. Notably, removing either the protein or gene component leads to only a minor performance degradation, with an average decrease of approximately 1% across all evaluation metrics. In contrast, removing the indirect association encoder causes a substantial decline, with AUC and AUPR decreasing by 12.72% and 13.73%, respectively. These findings indicate that gene- and protein-mediated signals provide partially complementary information, whereas indirect multi-hop associations contribute critical relational context that cannot be adequately captured by direct links or homogeneous similarities alone. The ablation study validates the robustness of HBGCN and underscores the importance of incorporating diverse biological interactions in DTI prediction.

**Table 7:** Result of ablation study.

Models	AUC	AUPR	F1-score	Precision	Recall
w/o protein	0.9476	0.9550	0.8767	0.8640	0.8998
w/o gene	0.9537	0.9454	0.8755	0.8485	0.9042
w/o indirect GCN	0.8312	0.8238	0.7493	0.7201	0.7040
HBGCN	**0.9584**	**0.9611**	**0.8912**	**0.8761**	**0.9072**

### Hyperparameter study

**Fig 6:**
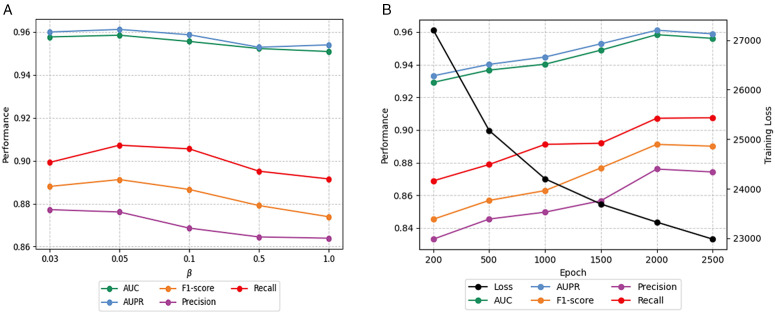
Hyperparameter sensitivity analysis of HBGCN. **(a)** The value of the loss weighted coefficient β. **(b)** The value of the training epoch. The prediction performance improves with the increased weight assigned to direct drug-disease associations and the expansion of training epochs.

We investigate the sensitivity of key hyperparameters and report the performance of HBGCN on the DTI prediction task under different parameter settings, as shown in Fig 6.

- The value of the loss weighting coefficient β: Since the loss of indirect drug–disease associations is significantly larger than that of direct associations, the available values of β are set to [0.03, 0.05, 0.1, 0.5, 1.0]. As shown in Fig 6(a), the performance of HBGCN decreases progressively as β increases, with the best performance obtained when β is set to 0.05. These findings indicate that appropriately reducing the contribution of indirect associations improves the accuracy of DTI prediction.
- The value of the training epoch: The second hyperparameter investigated is the number of training epochs. Fig 6(b) illustrates the variation of five evaluation metrics and the training loss under different epoch settings. As the number of epochs increases, the training loss decreases significantly, while all metrics gradually increase and eventually stabilize. Therefore, setting the epoch to 2000 is suitable for HBGCN.

## Conclusion

In this study, we propose a novel multimodal graph convolutional network, termed HBGCN, for drug–target interaction prediction. The proposed model exploits similarities among homogeneous entities and meta-paths among heterogeneous entities to characterize complex biological relationships. By integrating heterogeneous information, HBGCN captures higher-order semantic dependencies and overcomes the limitations of conventional methods that primarily rely on direct interactions. Through hierarchical graph propagation, the model iteratively aggregates and refines node representations, thereby promoting the convergence of pharmacologically related drugs and targets within a shared latent space. The interaction score is computed based on similarity to quantify the predicted association strength between drug–target pairs. Extensive experiments demonstrate that HBGCN outperforms existing methods on benchmark datasets. In drug repositioning tasks, HBGCN achieves improved performance over the best baseline, with a 5.43% increase in AUPR and a 7.4% increase in F1-score. Case studies demonstrate the potential of HBGCN to identify therapeutic drug candidates and elucidate underlying pharmacological mechanisms.

Despite these findings, the current framework derives initial molecular features exclusively from inter-entity relationships. Therefore, future work will focus on incorporating intra-entity elemental distribution patterns and multiview biological evidence within a unified learning objective to improve the biological coherence of learned embeddings. In addition, the current framework relies on sparse biological associations, which may introduce noise and limit the robustness of representation learning. To address these limitations, advanced graph learning techniques, such as link-based attributed graph clustering and hypergraph structure discovery, may further denoise sparse associations and provide principled structural priors for capturing informative features.

## Supporting information

S1 File Dataset. The dataset comprises four types of biological entities, including drugs, diseases, genes, and proteins, along with their corresponding interaction and association data.
(ZIP)
